# The circular RNA hsa_circ_0045800 serves as a favorable biomarker in pathogenesis of sjögren's syndrome

**DOI:** 10.1007/s10067-024-06999-0

**Published:** 2024-06-13

**Authors:** Hong Zhu, Yi Wang, Ge Wang, Yitong Ling, Jinhai Tian, Yan Zhou, Rong Zhu, Rui Wang, Ruixin Wang, Wenhui Zhang, Xiaoyu Zhang

**Affiliations:** 1 Department of Rheumatology, General Hospital of Ningxia Medical University, Yinchuan 750004, Ningxia, China; 2 The Second Department of Internal Medicine, Ningxia Gem Flower Hospital, Yinchuan 750006, Ningxia, China; 3 University of Health and Rehabilitation Sciences, Qingdao Municipal Hospital Group, Qingdao 266000, Shandong, China; 4 Department of Neurology, Jinan University First Afliated Hospital, Guangzhou 510000, Guangdong, China; 5 Biochip Center, General Hospital of Ningxia Medical University, Yinchuan 750004, Ningxia, China; 6 First Clinical Medical College of Ningxia Medical University, Yinchuan 750004, Ningxia, China; 7 Department of Intensive Care Unit Ward, Rizhao People’s Hospital, Rizhao, Shandong China; 8 Department of Infectious Diseases, The Third Affiliated Hospital of Sun Yat-Sen University, Guangzhou 510000, Guangdong, China; 9 Guangdong Provincial Key Laboratory of Liver Disease Research, Third Affiliated Hospital of Sun Yat-Sen University, Guangzhou 510000, Guangdong, China; 10 Central Laboratory, Rizhao People’s Hospital, Rizhao, China

**Keywords:** Hsa_circ_0045800, miR-1247-5p, Primary Sjögren’s syndrome, SMAD2

## Abstract

**Background:**

Circular RNAs (circRNAs) play various roles in the development of many autoimmune diseases. However, their expression profiles and specific function in Sjögren's Syndrome remains largely unknown.

**Objectives:**

We aimed to investigate circRNAs potential diagnostic value in primary Sjögren's syndrome (pSS) and contribution to the pathogenesis of pSS.

**Methods:**

This study included 102 subjects, 51 pSS patients and 51 healthy controls. The concentration of hsa_circ_0045800 was analyzed in peripheral blood mononuclear cells obtained from 51 pSS patients and 51 healthy controls by qRT-PCR. We established a receiver operating characteristic curve (ROC) to assess the biological diagnostic value of hsa_circ_0045800 for pSS. In addition, we analyzed the correlation between hsa_circ_0045800 and disease activity in Sjogren's syndrome. A differential analysis was also conducted on the concentration of hsa_circ_0045800 in patients in pSS patients before and after treatment. We studied the downstream mechanism of hsa_circ_0045800 through bioinformatics analysis and confirmed it using luciferase reporter gene assay.

**Results:**

We confirmed that the concentration of hsa_circ_0045800 was elevated 10.4-fold in peripheral blood mononuclear cells of pSS patients than in healthy controls (p = 0.00). In the pSS active disease group, the concentration of hsa_circ_0045800 is 2.5-fold higher compared to the pSS non-active disease group (p = 0.04). The concentration of hsa_circ_0045800 after treatment was decreased by 80% compared with that before treatment (p = 0.037), suggesting its utility as a potential marker for monitoring treatment efficacy. ROC curve analysis showed that the diagnostic value of hsa_circ_0045800 in pSS patients was significantly higher than that in healthy controls, with an area under the curve of 0.865, a sensitivity of 74%, and a specificity of 92%. The concentration of hsa_circ_0045800 is correlated with various clinical factors: the concentration of hsa_circ_0045800 is positively associated with age (r = 0.328, P = 0.019), oral dryness (r = 0.331, P = 0.017), while it is negatively correlated with HGB (r = -0.435, P = 0.001) and and hypothyroidism (r = -0.318, P = 0.023). Bioinformatics predictions and luciferase assays indicated that hsa_circ_0045800 acts as a molecular sponge for miR-1247-5p, with SMAD2 being a target gene of miR-1247-5p.

**Conclusion:**

Our study results show that hsa_circ_0045800 potentially contributes to the development and progression of pSS via the miR-1247-5p/SMAD2 pathway. Peripheral blood mononuclear cells are directly involved in the pathogenesis of pSS, and the discovery of hsa_circ_0045800 in peripheral blood mononuclear cells highlights its potential as a novel biomarker for disease activity and diagnosis in patients with pSS.

**Supplementary Information:**

The online version contains supplementary material available at 10.1007/s10067-024-06999-0.

**Table:** 

**Key Points** *• The concentration of hsa_circ_0045800 was higher in peripheral blood mononuclear cells of pSS patients.* *• Hsa_circ_0045800 promoted pSS progression through miR-1247-5p–SMAD2 axis.* *• Hsa_circ_0045800 is a potential biomarker for pSS.*	

## Introduction

Primary Sjogren's syndrome (pSS) is a chronic autoimmune disease that invades exocrine glands and lymphocyte proliferation. It is characterized by dry eyes and mouth caused by lacrimal gland and salivary gland dysfunction [1–3], which mainly affects middle-aged women, with a male-to-female ratio of 1: 9 [4, 5]. At present, the pathogenesis of pSS is unknown, and many factors such as heredity, infection and environment are involved in the pathogenesis [6]. In recent years, people have been trying to find reliable biomarkers to improve the diagnosis algorithm and new treatment scheme of pSS patients.

CircRNA is a newly discovered single-stranded circRNA molecule, which is formed by the reverse shear of the downstream sequence of the precursor mRNA (the 3' end) and the upstream RNA sequence (the 5' end) to form a special ring structure. CircRNA has neither 5' to 3' polarity nor a polyadenylation tail, so it is not easy to be degraded by exonucliase [7]. At present, circRNA has been found to have various types, rich content, long half-life and good stability [8, 9]. Recent studies have shown that its cis-regulation of parent genes [7], sponge miRNA [10], binding to proteins and forming functional complexes [11], translation proteins [12] and other roles play key roles in eukaryotic cells. Studies have been conducted on the relationship between circRNA and various diseases, such as tumor, cardiovascular disease, nervous system disease, autoimmune disease, etc. [13–16]. Therefore, circRNA can be used as a biomarker as a potentially valuable diagnostic tool for pSS disease. There are few studies on the role of circRNA in the pathogenesis of pSS.

In our previous research, We have explored the clinical correlation of circRNA and pSS and found that the expression of hsa_circ_0045800 in peripheral blood of patients with pSS was significantly increased by screening with high-throughput gene chip [17]. It may be used as a diagnostic marker for pSS. In this study, a novel circRNA, hsa_circ_0045800, was identified in peripheral blood mononuclear cells, that was associated positively with pSS disease activity. Hsa_circ_0045800 exhibited notable specificity and sensitivity, suggesting strong potential as a diagnostic biomarker for pSS. Furthermore, we explored that hsa_circ_0045800 could act as a sponge for hsa-miR-1247-5p to regulate SMAD2 expression in the progression of pSS.

## Methods

### Participant information

This study involved 51 pSS patients admitted to the Department of Rheumatology at the General Hospital of Ningxia Medical University between June 2018 and June 2021. These patients were diagnosed with primary Sjögren's syndrome (pSS) based on the 2002 American-European Consensus Group (AECG) standards combined with the 2016 American College of Rheumatology (ACR)/European League Against Rheumatism (EULAR) classification criteria [18]. All patients had complete case data and had signed informed consent forms.

The exclusion criteria for the case group included: not meeting the diagnostic criteria for primary Sjögren's syndrome (pSS), the coexistence of other autoimmune diseases, the presence of head, neck tumors, and lymphomas, concomitant hematologic disorders (considering pSS can involve the hematologic system), recent severe infections (indicated by elevated inflammatory factors), severe cardiac or hepatic failure (evidenced by abnormal liver and kidney functions), and recent use of cholinergic drugs (which can cause symptoms of dry mouth).The control group comprised 51 healthy individuals who underwent medical examinations at the General Hospital of Ningxia Medical University during the same period. The inclusion criteria for the control group were: not meeting the diagnostic criteria for primary Sjögren's syndrome (pSS), no personal or immediate family history of autoimmune diseases, no use of steroids, immunosuppressants, or similar medications including cyclophosphamide, methotrexate, leflunomide, azathioprine, and cyclosporine in the past three months, and being free from infections, tumors, and communicable diseases.

The study received approval from the Ethics Committee of the General Hospital of Ningxia Medical University [2018–099, 2020–313], and all participating researchers had signed informed consent forms.

### Specimen collection and RNA isolation

The peripheral blood (5 ml) of the participants was collected by EDTA anticoagulation vacuum tube, and the blood was stored in a specimen storage box at 4℃. Within 4 h after collection of peripheral blood samples, peripheral blood mononuclear cells were isolated from pSS patients and healthy controls using human peripheral blood lymphocyte separation medium (Biyuntian, China). Total RNA was extracted by Batek RNA extraction kit RP4002 according to the in Total RNA was extracted by Batek RNA extraction kit RP4002 according to the instructions, and RNA concentration and purity were measured by Nanodrop2000( Thermo Scientific, USA). The isolated total RNA was stored at-80 C or immediately used for reverse transcription.

### Real-time quantitative reverse transcription PCR (qRT-PCR)

The prepared total RNA was reverse transcribed by Primescript RT kit (Takara Bio, Japan) according to the product specification to obtain total cDNA, which was used as template and β-actin gene as internal reference. Using the Lightcycler 480 System, the reaction conditions were kept at 95℃ for 3 s, and the amplification was carried out after 40 cycles of denaturation at 95℃ for 5 min and denaturation at 60℃ for 30 s. The experimental results showed that the difference of CT value (the number of fractional cycles of fluorescence exceeding a given national value) was less than 0.5, and the concentration of circ_0045800 was analyzed by 2^−△△Ct^ method, and the experiment was repeated three times. Primers used in this study were hsa_circ_0045800 upstream primer 5'-TTTACTTGCAGTGAAGCGCC-3', downstream primer 5'-GCTTCGCTCTTGGTTTTGGA-3', and reference β-actin upstream primer 5 '-CCACGGCTGCTTCCAGCTCC-3', downstream primer 5 '-GGACTCCATGCCCAGGAAGGAA-3'.

### Cell transfection

The miR-1247-5p mimics were designed by HanYi Biosciences lnc (Guangzhou, China) NC for mimics used as a corresponding negative control. Additionally, the luciferase reporter plasmids, which included both the wild-type and mutant sequences of hsa_circ_0045800 and the SMAD2 3′-UTR were supplied by HanYi Biosciences lnc (Guangzhou, China). These plasmids, together with the miRNA mimics, were transfected into HEK293T cells using the transfection agent Lipofectamine 2000, provided by Invitrogen in the USA. Following a 24–48 h incubation period post-transfection, the cells were harvested for further experimental analysis. The mimics sequences for miR-1247-5p are listed below: ACCCGUCCCGUUCGUCCCCGGA.

### Dual-luciferase reporter assay

We used luciferase reporter gene method to identify the binding of selected RNA molecules. The luciferase reporter plasmids of wild sequence hsa_circ_0045800, SMAD2 3 ′-UTR and mutant sequence hsa_circ_0045800, SMAD2 3 ′-UTR were constructed. Subsequently, 2 × 10^5^ HEK293T cells were inoculated into a 24-well plate and co-transfected with a mixture of 1 μg luciferase reporter plasmids and miRNA mimics. After incubation for 48 h, the activities of luciferase and reninase were quantitatively determined by double luciferase detection system. Detection system (Promega, Madison, USA).

### Statistical analysis

Statistical analysis was performed using GraphPad Prism v5.0 software (GraphPad software, Inc.) and SPSS 25.0 software (SPSS, Inc. to analyze circRNAs expression differences between the two groups using Student's t test or nonparametric Mann–Whitney test. Wilcoxon rank sum test was used for paired samples. Spearman rank correlation analysis was used to analyze the correlation among parameters. The diagnostic value of circRNAs was evaluated by receiver operating characteristic (ROC) curve. P < 0.05 was considered to be statistically significant.

## Results

### The characteristics of the study population

In this study, we collected 51 patients with pSS including 47 females and 4 males, aged from 21 to 77 years old, and 51 healthy controls including 42 females and 9 males, aged from 20 to 68 years old. There was no significant difference in age or sex between pSS patients and healthy controls (Table 1).

**Table 1:** General data analysis of this study population

	pSS patient	Healthy control	*t/2 value*	*P value*
Age (years)	49.9 ± 13.73	46.0 ± 11.99	1.068	0.288
Sex(male/female)	4/47	9/42	2.210	0.137

There was no significant difference in age or sex between the pSS patients and healthy controls

### Identification of hsa_circ_0045800 concentration difference in peripheral blood mononuclear cells between pSS patients and control controls.

**Fig. 1:**
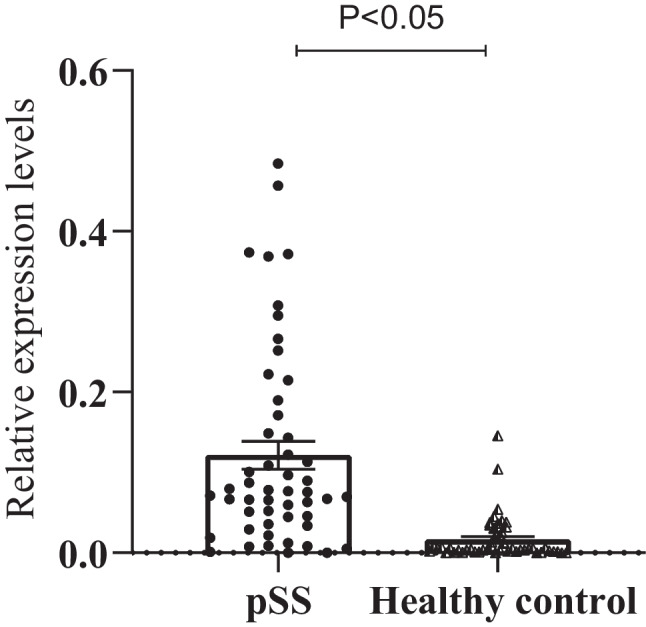
The concentration of hsa_circ_0045800 in pSS patients and healthy controls. By determining the concentration of hsa_circ_0045800, we found that hsa_circ_0008301 expression concentration was increased in patients with pSS when compared with healthy controls(P < 0.05)

The concentration of hsa_circ_0045800 in peripheral blood mononuclear cells of pSS patients was higher 10.4-fold than in the healthy controls (p = 0.000) (Fig. 1, Supplement Table 1).

### Potential biological value of hsa_circ_0045800 in patients with pSS.

**Fig. 2:**
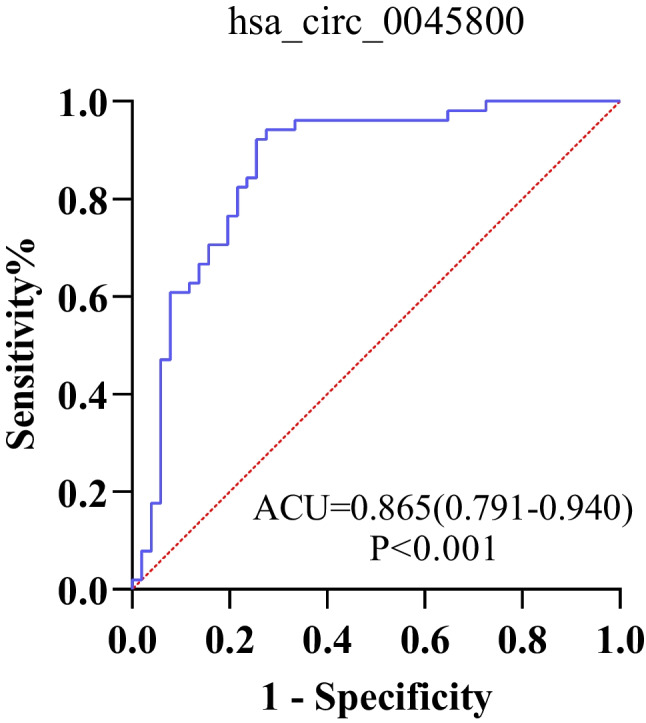
The ROC curve assessing the diagnostic potential of hsa_circ_0045800 concentration for pSS. The ROC curve analysis on the concentration of hsa_circ_0045800 in pSS patients and the health controls that hsa_circ_0045800 has high diagnostic efficiency in pSS patients

ROC curve analysis results for hsa_circ_0045800 showed that the ROC curve area (AUC) was 0.865 (95%CI = 0.791–0.940, P < 0.05), the sensitivity was 74%, and the specificity was 92% (Fig. 2).

### Differential concentration of hsa_circ_0045800 in patients with pSS.

We compared the concentration of hsa_circ_0045800 in different gender, age and course of disease in pSS patients. Hsa_circ_0045800 concentration was higher 2.6-fold in patients aged 50 years and above than in patients aged less than 50 years (p = 0.032) (Table 2, Supplement Table 1). There was no significant difference in the concentration of hsa_circ_004580 between the sexes and the course of disease (Table 2).

**Table 2:** Comparison of hsa_circ_0045800 concentration in different gender, age, ESSDAI, treatment and course of disease in pSS patients

	n	hsa_circ_0045800 *M*(*P*25,*P*75)	*Z value*	*P value*
Sex			-1.051	0.314
female	47	0.077 (0.036, 0.190)		
male	4	0.057 (0.029, 0.076)		
Age (years)			-2.149	0.032*
≥ 50	22	0.103 (0.066, 0.266)		
< 50	29	0.063 (0.026, 0.107)		
Disease duration(years)			-0.321	0.748
≥ 10	11	0.097 (0.019, 0.190)		
< 10	40	0.074 (0.038, 0.166)		
ESSDAI			-2.053	0.016*
≥ 5	43	0.088 (0.052, 0.185)		
< 5	8	0.029 (0.009, 0.066)		
Untreated			-2.075	0.037*
Yes	15	0.080 (0.040, 0.255)		
No	15	0.034 (0.010, 0.105)		

The difference in the concentration of hsa_circ_0045800 among PSS patients aged 50 or above, with ESSDAI greater than or equal to 5, and whether they received treatment, has statistical significance

**Fig. 3:**
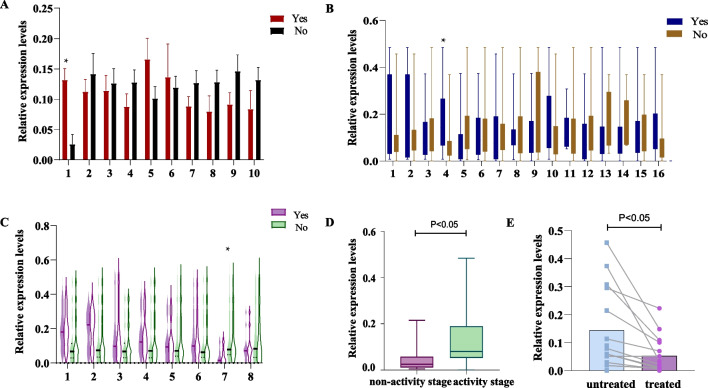
The concentration of hsa_circ_0045800 in pSS patients is different. A, The concentration of hsa_circ_0045800 was varied in clinical manifestations. 1 Oral dryness (Yes: n = 46 No: n = 5), 2 Ocular dryness (Yes: n = 35 No: n = 16), 3 Dental damage (Yes: n = 19 No: n = 32), 4 Skin dryness (Yes: n = 8 No: n = 43), 5 Fatigue (Yes: n17 No: n = 34), 6 Skin purpura (Yes: n = 7 No: n = 44), 7 Joint pain (Yes: n = 7 No: n = 44), 8 Lymph node enlargement (Yes: n = 7 No: n = 44), 9 Raynaud's phenomenon (Yes: n = 7 No: n = 44), 10 Morning stiffness (Yes: n = 11 No: n = 40). * indicates P < 0.05, which is statistically significant. B, The concentration of hsa_circ_0045800 were differ from laboratory indexes in pSS patients. 1. WBC < 3.5 * 10 ^ 9 / L (Yes: n = 36 No: n = 15), 2. NEUT < 1.5 * 10 ^ 9 / L (Yes: n = 41 No: n = 10), 3. LYM < 1.0 * 10 ^ 9 / L (Yes: n = 40 No: n = 11), 4.HGB < 120 g/L (Yes: n = 28 No: n = 23), 5.PLT < 100*10^9/L (Yes: n = 35 No: n = 16), 6. CRP acuity 2.87 mg/L (Yes: n = 14 No: n = 37), 7.ESR ≥ 50 mm/h (Yes: n = 15 No: n = 36), 8.IL6 ≥ 7 pg/ml (Yes: n = 9 No: n = 42), 9.IgG ≥ 16 g/L (Yes: n = 43 No: n = 8), 10. C3 < 0.9 g/L (Yes: n = 37 No: n = 14), 11.C4 < 0.1 g/L (Yes: n = 43 No: n = 8), 12.RF ≥ 19 IU/ml (Yes: n = 15 No: n = 36), 13.AbSSA(52) + (Yes: n = 44 No: n = 7), 14.AbSSA(60) + (Yes: n = 45 No: n = 6), 15.AbSSB + (Yes: n = 41 No: n = 10), 16. Anti-nuclear antibody tite ≥ 1:320 (Yes: n = 937 No: n = 14). * indicates P < 0.05, which is statistically significant.) C, The concentration of hsa_circ_0045800 was different in pSS patients with complications. 1 Hypertension (Yes: n = 9 No: n = 41), 2 Coronary heart disease (Yes: n = 8 No: n = 42), 3 Hyperlipidemia (Yes: n = 25 No: n = 25), 4 Hyperuricemia (Yes: n = 11 No: n = 39), 5 Fatty liver (Yes: n = 6 No: n = 45), 6 Interstitial lung disease (Yes: n = 23 No: n = 27), 7 Hypothyroidism (Yes: n = 6 No: n = 45), 8 Chronic gastritis (Yes: n = 17 No: n = 33). * indicates P < 0.05, which is statistically significant. D, There were differences in the concentration of hsa_circ_0045800 in different disease activity stage of pSS patients. E, The concentration of hsa_circ_0045800 after treatment was decreased compared with that before treatment

Subsequently, the concentration of hsa_circ_0045800 with different clinical manifestations were compared in pSS patients. The concentration of hsa_circ_0045800 was higher 4.7-fold in patients with oral dryness symptoms than in patients without oral dryness symptoms (P = 0.016). (Fig. 3A, Supplement Table 1)*.* However, the concentration of hsa_circ_0045800 showed no statistical significance in the presence or absence of ocular dryness, tooth damage, dry skin, fatigue, purpura, joint pain, lymph node enlargement, Raynaud's phenomenon, and morning stiffness (Fig. 3A, Supplement Table 2).

We also compared the concentration of hsa_circ_0045800 in different laboratory test indicators in pSS patients. The concentration of hsa_circ_0045800 in patients with HGB < 120 g/L was higher 3.4-fold than that in patients with HGB ≥ 120 g/L (p = 0.003) (Fig. 3B, Supplement Table 1). In the conducted study, a range of experimental indicators in pSS patients, including White Blood Cell count (WBC), Neutrophil count (NEUT), Lymphocyte count (LYM), Platelet count (PLT), C-Reactive Protein (CRP), Erythrocyte Sedimentation Rate (ESR), Interleukin 6 (IL6), Immunoglobulin G (IgG), Complement C3 and C4, Rheumatoid Factor (RF), anti-SSA (52) and (60) antibodies, anti-SSB antibody, and Antinuclear Antibody titers, were divided into different level groups based on specific thresholds. The results showed that regardless of the group, there were no statistically significant differences in the concentration of hsa_circ_0045800. This indicates that the concentration of hsa_circ_0045800 is not influenced by these biochemical and immunological parameters, demonstrating its independence from these indicators (Fig. 3B, Supplement Table 3).

The concentration of hsa_circ_0045800 was decreased by 83% in pSS patients with hypothyroidism than in patients without hypothyroidism (p = 0.022) (Fig. 3C, Supplement Table 1). There was no significant difference in hsa_circ_0045800 concentration among pSS patients with hypertension, coronary heart disease, hyperlipidemia, hyperuricemia, fatty liver, pulmonary interstitial disease, and chronic gastritis (Fig. 3C, Supplement Table 4).

According to the pSS disease activity index integral criteria to judge activity degree of patient's disease, [18] ESSDAI score of 5 or more cases for activity, ESSDAI score < 5 points for non-activity. In this study, the ESSDAI score of pSS patients was 10.29 ± 5.565 points. There were 43 pSS patients in the disease active group (11.72 ± 5.378 points) and 8 pSS patients in the non-disease active group (2.75 ± 1.165 points). The concentration of hsa_circ_0045800 in the disease active group was higher 2.5-fold than that in the non-disease active group (p = 0.04) (Fig. 3D, Table 2, Supplement Table 1).

Hsa_circ_0045800 concentration after treatment in pSS patients was decreased by 80% compared with that before treatment (p = 0.037) (Fig. 3E, Table 2, Supplement Table 1).

### Correlation analysis of hsa_circ_0045800 concentration with clinical features and disease activity in patients with pSS

The concentration of hsa_circ_0045800 was positively correlated with age (r = 0.328, P = 0.019), oral dryness (r = 0.331, P = 0.017). On the contrary, it was negatively correlated with HGB (r = -0.435,P = 0.001) and and hypothyroidism (r = -0.318, P = 0.023). And it had no correlation with such clinical features including sex, disease duration (month), ocular dryness, tooth damage, dry skin, fatigue, purpura, joint pain, lymph node enlargement, Raynod's phenomenon, morning stiffness, WBC, NEUT, LYM, PLT, CRP, IL-6, IgG, C3, C4, RF, AbSSA(52), AbSSA(60), AbSSB, ESSDAI, antinuclear antibody titer, combined hypertension, coronary heart disease, hyperlipidemia, hyperuricemia, fatty liver, interstitial lung disease, chronic gastritis (Table 3).

**Table 3:** The association between the concentration of hsa_circ_0045800 and the clinical characteristics as well as disease activity of patients afflicted with pSS

Index	R value	*P* value
Sex (male/female)	0.17	0.228
Age (years)	0.328	0.019*
Disease duration(years)	0.005	0.971
Oral dryness	0.331	0.017*
Ocular dryness	-0.077	0.593
Tooth damage	0.135	0.347
Skin dryness	-0.041	0.774
Fatigue	0.138	0.335
Purpura	0.123	0.392
Joint pain	-0.209	0.144
Lymph node enlargement	0.006	0.967
Raynaud's phenomenon	-0.110	0.448
Morning stiffness	-0.202	0.159
WBC(*10^9/L)	-0.087	0.550
NEUT(*10^9/L)	-0.136	0.348
LYM(*10^9/L)	0.039	0.790
HGB(120 g/L)	-0.435	0.001*
PLT(100*10^9/L)	-0.143	0.610
CRP(mg/L)	-0.338	0.233
ESR(mm/h)	0.606	0.058
IL-6(pg/ml)	0.300	0.432
IgG(g/L)	0.053	0.735
C3(g/L)	0.181	0.553
C4(g/L)	0.392	0.383
RF(IU/ml)	-0.019	0.946
Anti-Ro-52 body	-0.161	0.261
Anti-Ro-60 body	-0.217	0.129
Anti-SSB body	-0.170	0.228
Antinuclear antibodies	0.196	0.176
ESSDAI	0.656	0.000*
Index	r value	P value
Hypertension	0.250	0.079
Coronary heart disease	0.177	0.216
Hyperlipemia	-0.034	0.811
Hyperuricemia	0.095	0.508
Fatty liver	0.095	0.513
Interstitial lung disease	-0.059	0.680
Hypothyroidism	-0.318	0.023*
Chronic gastritis	-0.147	0.305

The concentration of hsa_circ_0045800 was positively correlated with age (r = 0.328, P = 0.019), oral dryness (r = 0.331, P = 0.017). It was negatively correlated with HGB (r = -0.435,P = 0.001) and hypothyroidism (r = -0.318, P = 0.023)

### Hsa_circ_0045800 acts as a miRNA sponge for miR-1247-5P

**Fig. 4:**
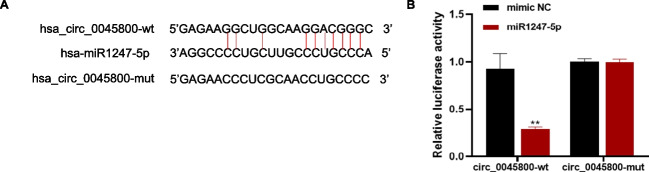
Hsa_circ_0045800 functions as a sponge for miR-1247-5P. **A**, The binding sites between hsa_circ_0045800 and miR-1247-5P were predicted by bioinformatics analysis. **B**, The binding sites between hsa_circ_0045800 and miR-1247-5P were verified by a double luciferase reporter gene assay

Our investigation focused on exploring the potential miRNA targets of hsa_circ_0045800, a circular RNA known to function as a miRNA sponge. Using bioinformatics tools such as CircInteractome and circBank, we conducted an analysis to identify complementary miRNAs. Interestingly, hsa-miR-1247-5p emerged as the top candidate with a perfectly matched binding sequence to hsa_circ_0045800 (Fig. 4A). To validate the direct interaction between hsa_circ_0045800 and hsa-miR-1247-5p, we performed a dual-luciferase reporter assay in HEK293T cells. The experimental results indicated a significant decrease in relative luciferase activity when co-transfecting hsa_circ_0045800 WT with hsa-miR-1247-5p mimics compared to the control group. Conversely, co-transfection of hsa_circ_0045800 MUT with hsa-miR-1247-5p mimics did not induce any change in relative luciferase activity compared to the control group (Fig. 4B). Overall, these findings provide compelling evidence supporting the direct targeting of hsa-miR-1247-5p by hsa_circ_0045800.

### SMAD2 is the target gene of miR-1247-5P

**Fig. 5:**
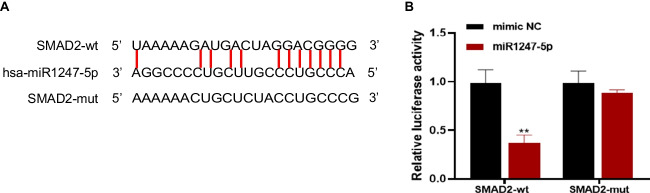
SMAD2 is the target gene of miR-1247-5P. A, The binding sites between SMAD2 and miR-1247-5P were predicted by bioinformatics analysis. B, The binding sites between SMAD2 and miR-1247-5P was verified by double luciferase reporter gene assay

We utilized bioinformatics tools (targetScan and ENCORI) to search for potential target genes of hsa-miR-1247-5p. To further investigate the interaction between hsa-miR-1247-5p and SMAD2, we introduced plasmids containing SMAD2-3’ UTR-WT and SMAD2-3’ UTR-MUT into HEK293T cells. Interestingly, the luciferase activity was significantly reduced in cells co-transfected with SMAD2-3’ UTR-WT and hsa-miR-1247-5p mimics compared to the control group (Fig. 5).

## Discussion

In the progression of various autoimmune disorders, circRNAs exert their biological influence through a variety of mechanisms [8]. Mounting evidence has demonstrated that circRNAs may play a pivotal role in governing gene expression and transcription by functioning as microRNA (miRNA) reservoirs, impacting cell viability and proliferation through interaction with RNA binding proteins (RBPs), and enhancing mRNA stability by forming RNA–protein complex duplex structures [11, 14, 15]. In systemic lupus erythematosus (SLE), the expression level of circLOC101928570 experiences significant downregulation, which exhibits correlation with the systemic lupus erythematosus disease activity index. Functioning as a molecular reservoir for miR-150-5p, circLOC101928570 alleviates the inhibitory effect of its target gene, c-myb [19]. Moreover, Circ_0088194 stimulates the invasion and migration of fibroblast-like synovial cells in rheumatoid arthritis via the miR-766-3p/MMP2 axis [20]. Nonetheless, only a limited number of studies have elucidated the role of circRNAs in primary Sjögren's syndrome (pSS). In our present study, we initially identified a pivotal circRNA (circURE2O, circBase ID: hsa_circ_0045800) in pSS. The principal findings indicated that it had substantial elevated concentration in peripheral blood mononuclear cells of pSS patients, which closely correlate with multiple clinical features and disease activity.

We conducted a thorough analysis of the association between hsa_circ_0045800 and the clinical characteristics of primary Sjögren's syndrome (pSS). The concentration of hsa_circ_0045800 in pSS patients with activity stage was notably higher than that with non-activity stage, displaying a positive correlation with the European League Against Rheumatism Sjögren's Syndrome Disease Activity Index (ESSDAI). Notably, the concentration of hsa_circ_0045800 decreased significantly after treatment. Additionally, the concentration of hsa_circ_0045800 exhibited a decline in pSS patients with hypothyroidism, demonstrating a negative correlation with this condition. Interestingly, the concentration of hsa_circ_0045800 was higher in patients with hemoglobin levels below 120 g/L, displaying a negative correlation with hemoglobin (HGB). This study revealed that has_circ_0045800 possesses high sensitivity and specificity, suggesting its potential as a valuable biomarker associated with the diagnosis of pSS. This discovery opens up new possibilities for early screening and differential diagnosis of pSS. Future investigations could explore the combination of hsa_circ_0045800 with established pSS biomarkers to enhance diagnostic accuracy and reliability. The parental gene of hsa_circ_0045800, UBE2O (ubiquitin binding enzyme E2O), operates as an E3-independent E2 (i.e., E2/E3 heterosynthase), enabling direct ubiquitination of various substrates [21]. GomeZ-Martin et al. demonstrated the significant upregulation of the ubiquitination signaling pathway in pSS patients [22]. UBE2O plays a vital role in diverse cellular processes, encompassing cell cycle regulation, protein quality control, DNA damage repair, cellular development, and differentiation, among other biological processes [23–27]. This study further explored the molecular mechanism of hsa_circ_0045800 in pSS, revealing its capability to promote pSS progression by targeting miR-1247-5p and SMAD2.

MicroRNAs (miRNAs) are diminutive noncoding, unpaired RNAs that govern gene expression and impact numerous cellular processes such as proliferation, differentiation, and apoptosis [28, 29]. MiR-1247 consists of miR-1247-5p and miR-1247-3p [30, 31]. Studies have revealed that miR-1247 may act as a key regulator of cartilage function, with substantial effects on cartilage matrix production from human chondrocytes [30]. It directly targets the cartilage master regulator gene SOX9 through a deeply conserved region within its coding sequence [30]. Jennifer Liang et al. demonstrated that miR-1247 functions as an innovative tumor suppressor by inhibiting MYCBP2 in methylated colon cancer. The role of miR-1247 in pSS remains unknown [31]. Using database analysis, we identified binding sites for hsa_circ_0045800 and miR-1247-5p, which were subsequently confirmed by luciferase assay. This discovery suggests that hsa_circ_0045800 may act as a molecular reservoir for miR-1247-5p, regulating its downstream target genes involved in the progression of pSS. Furthermore, using luciferase reporter assays, we investigated the potential binding of miR-1247-5p to SMAD2, revealing a greatly conserved region within the encoding sequence of SMAD2 as the binding site, rather than its 3’ UTR.

Recognized as a member of the SMAD family, SMAD2 functions as an intracellular signal transducer and downstream transcriptional regulator of transforming growth factor-β (TGF-β) [32, 33]. The involvement of SMAD2 spans fetal development, inflammatory responses, tissue differentiation, and even tumorigenesis. Transforming growth factor (TGF)-β, a multifaceted cytokine, governs cell growth and differentiation, extracellular matrix deposition, fibrosis, and installation [34]. TGF-β1, specifically, plays a pivotal role in initiating fibrosis, commonly observed during chronic stages of inflammatory diseases, which detrimentally affect normal organ function [35, 36]. The biological effects of TGF-β1 signaling are mediated through the TGF-β/SMAD/Snail pathway, thereby exerting a significant pathogenic influence on various fibrotic disorders [37]. However, its implications in Sjögren's syndrome (SS), a chronic autoimmune disease, have received limited investigation. Prior studies indicate that circRNAs can modulate the TGF-β/SMAD pathway in breast cancer (BCa) [38], with circRIP2 notably expediting BCa progression via the miR-1305/TGF-β2/smad3 pathway [35, 37, 38]. In this study, a compelling finding emerged wherein hsa_circ_0045800 was found to regulate SMAD2 by suppressing miR-1247-5p, thereby further substantiating the pivotal role of circRNAs in modulating the activation of the TGF-β/SMAD pathway.

However, certain limitations were also encountered. For instance, it is possible that hsa_circ_0045800 targets additional miRNAs and modulates alternative downstream pathways, which necessitates further exploration of its underlying mechanisms. Noncoding RNAs, such as micro-RNAs, exhibit minimal specificity towards a single disease, posing challenges in utilizing a solitary molecule for disease diagnosis or activity assessment. Instead, circRNAs offer potential as an adjunctive diagnostic tool, distinct from conventional laboratory serological markers and inflammation-related parameters, thereby warranting consideration.

In conclusion, we have unveiled previously unknown circRNA, hsa_circ_0045800, in peripheral blood mononuclear cells of pSS. Peripheral blood mononuclear cells are a heterogeneous group of cells, which play a key role in the immune response and are directly involved in the pathogenesis of pSS. Mechanistically, hsa_circ_0045800 may be involved in the development of pSS through miR-1247-5p/SMAD2. These findings hold promise for clinical application, underscoring the need for comprehensive investigation into the biological roles and molecular mechanisms of circRNAs.

### Supplementary Information

Below is the link to the electronic supplementary material.

Supplementary file1 (DOCX 17 KB)

Supplementary file2 (DOCX 16 KB)

Supplementary file3 (DOCX 17 KB)

Supplementary file4 (DOCX 13 KB)

## Data Availability

The dataset analyzed in this study is available through request from the corresponding author at nxzhuh@126.com.
