# Supplementary material for: The circular RNA hsa_circ_0045800 serves as a favorable biomarker in pathogenesis of sjögren's syndrome
Source: Clin Rheumatol. 2024 Jun 13;43(8):2585–94. doi: 10.1007/s10067-024-06999-0 (PMC11269352; doi:10.1007/s10067-024-06999-0)
Supplement: Supplementary file 1 — Supplementary file1 (DOCX 17 KB) [file 10067_2024_6999_MOESM1_ESM.docx]

**Supplyment Table 1**

| Index | n | hsa_circ_0045800  M（P25,P75） | P value | Average △Ct | Average △△Ct | 2^-△△Ct^（Fold change） |
| --- | --- | --- | --- | --- | --- | --- |
| PSS  Y  N |  |  | 0.000 |  |  |  |
|  | 51 | 0.076（0.036，0.171） |  | 4.118 | -3.373 | 10.361 |
|  | 51 | 0.006（0.018，0.026） |  | 7.491 | 0 | 1 |
| Oral dryness  Y  N |  |  | 0.016 |  |  |  |
|  | 46 | 0.034（0.027，0.056） |  | 3.898 | -2.4 | 4.731 |
|  | 5 | 0.009（0.007，0.054） |  | 6.140 | 0 | 1 |
| Hypothyroidism  Y  N |  |  | 0.022 |  |  |  |
|  | 6 | 0.015（0.006，0.075） |  | 6.335 | 2.513 | 0.175 |
|  | 45 | 0.078（0.049，0.202） |  | 3.822 | 0 | 1 |
| HGB<120  Y  N |  |  | 0.003 |  |  |  |
|  | 23 | 0.061（0.024，0.085） |  | 3.147 | -1.767 | 3.404 |
|  | 28 | 0.143（0.067，0.266） |  | 4.915 | 0 | 1 |
| Untreated  Y  N |  |  | 0.037 |  |  |  |
|  | 15 | 0.079(0.040, 0.255) |  | 3.193 | -2.357 | 5.124 |
|  | 15 | 0.033(0.010, 0.105) |  | 5.551 | 0 | 1 |
| ESSDAI ≧5  Y  N |  |  | 0.016 |  |  |  |
|  | 43 | 0.088（0.052，0.185） |  | 3.912 | -1.309 | 2.478 |
|  | 8 | 0.029（0.009，0.066） |  | 5.221 | 0 | 1 |
| Age (year)  ≥50  <50 |  |  | 0.032 |  |  |  |
|  | 22 | 0.103（0.066，0.266） |  | 3.321 | -1.400 | 2.639 |
|  | 29 | 0.063（0.026，0.107） |  | 4.721 | 0 | 1 |
