# Supplementary material for: The circular RNA hsa_circ_0045800 serves as a favorable biomarker in pathogenesis of sjögren's syndrome
Source: Clin Rheumatol. 2024 Jun 13;43(8):2585–94. doi: 10.1007/s10067-024-06999-0 (PMC11269352; doi:10.1007/s10067-024-06999-0)
Supplement: Supplementary file 2 — Supplementary file2 (DOCX 16 KB) [file 10067_2024_6999_MOESM2_ESM.docx]

**Supplyment Table 2**

| Index | n | hsa_circ_0045800  *M*（P25,P75） | Z value | *P* value |
| --- | --- | --- | --- | --- |
| Oral dryness |  |  | -2.344 | 0.016* |
| Yes | 46 | 0.034（0.027，0.056） |  |  |
| No | 5 | 0.009（0.007，0.054） |  |  |
| Ocular dryness |  |  | -0.541 | 0.589 |
| Yes | 35 | 0.056（0.030，0.147） |  |  |
| No | 16 | 0.093（0.027，0.244） |  |  |
| Tooth damage |  |  | -0.950 | 0.342 |
| Yes | 19 | 0.084（0.066，0.150） |  |  |
| No | 32 | 0.049（0.017，0.082） |  |  |
| Skin dryness |  |  | -0.292 | 0.785 |
| Yes | 8 | 0.074（0.042，0.136） |  |  |
| No | 43 | 0.076（0.042，0.217） |  |  |
| Fatigue |  |  | -0.973 | 0.331 |
| Yes | 17 | 0.113（0.042，0.302） |  |  |
| No | 34 | 0.076（0.031，0.146） |  |  |
| Skin Purpura |  |  | -0.274 | 0.799 |
| Yes | 7 | 0.088（0.058，0.324） |  |  |
| No | 44 | 0.074（0.039，0.166） |  |  |
| Joint pain |  |  | -0.611 | 0.541 |
| Yes | 26 | 0.070（0.040，0.136） |  |  |
| No | 25 | 0.090（0.039，0.233） |  |  |
| Lymph node enlargement |  |  | -0.042 | 0.978 |
| Yes | 7 | 0.076（0.063，0.113） |  |  |
| No | 44 | 0.032（0.019，0.052） |  |  |
| Raynaud's phenomenon |  |  | -0.769 | 0.459 |
| Yes | 7 | 0.063（0.022，0.080） |  |  |
| No | 44 | 0.032（0.009，0.052） |  |  |
| Morning stiffness |  |  | -1.417 | 0.157 |
| Yes | 11 | 0.066（0.034，0.087） |  |  |
| No | 40 | 0.032（0.032，0.076） |  |  |
