# Supplementary material for: The circular RNA hsa_circ_0045800 serves as a favorable biomarker in pathogenesis of sjögren's syndrome
Source: Clin Rheumatol. 2024 Jun 13;43(8):2585–94. doi: 10.1007/s10067-024-06999-0 (PMC11269352; doi:10.1007/s10067-024-06999-0)
Supplement: Supplementary file 3 — Supplementary file3 (DOCX 17 KB) [file 10067_2024_6999_MOESM3_ESM.docx]

**Supplyment Table 3**

| Index | n | hsa_circ_0045800  *M*（P25,P75） | Z value | *P* value |
| --- | --- | --- | --- | --- |
| WBC（*10^9/L）  ≥3.5  <3.5  NEUT#（*10^9/L）  ≥1.5  <1.5  LYM#（*10^9/L）  ≥1.0  <1.0  HGB（g/L）  ≥120  <120  PLT（*10^9/L）  ≥100  <100  CRP（mg/L）  ≥2.87  <2.87  ESR（mm/h）  ≥50  <50  IL6（pg/ml）  ≥7  <7  IgG（g/L）  ≥16  <16  C3（g/L）  ≥0.9  <0.9  C4（g/L）  ≥0.1  <0.1  RF（IU/ml）  ≥19  <19  Anti-Ro-52 body  +  —  Anti-Ro-60 body  +  Index | 36  15  41  10  40  11  28  23  35  16  14  37  15  36  9  42  43  8  37  14  43  8  15  36  44  7  45  n | 0.076（0.052，0.113）  0.143（0.032，0.368）  0.076（0.052，0143）  0.066（0.019，0369）  0.076（0.046，0.215）  0.077（0.032，0.143）  0.061（0.024，0.085）  0.143（0.067，0.266）  0.078（0.046，0.171）  0.067（0.012，0.190）  0.088（0.027，0.184）  0.069（0.045，0.185）  0.109（0.024，0.249）  0.071（0.045，0.147）  0.076（0.049，0.126）  0.077（0.040，0.202）  0.076（0.041，0.157）  0.077（0.039，0.415）  0.077（0.035，0.160）  0.071（0.049，0.249）  0.077（0.036，0.190）  0.071（0.052，0.143）  0.077（0.047，0.204）  0.068（0.011，0.160）  0.076（0.036，0.149）  0.101（0.067，0.295）  0.072（0.034，0.147）  hsa_circ_0045800  *M*（P25,P75） | -0.561  -0.388  -0.246  -2.991  -0.857  -0.259  -0.387  -0.063  -0.159  -0.718  -0.181  -0.907  -1.132  -1.523  Z value | 0.575  0.698  0.806  0.003*  0.391  0.795  0.699  0.960  0.886  0.472  0.869  0.364  0.270  0.134  *P* value |
| —  Anti-SSB body  +  —  Antinuclear antibodies  ≥1：320  <1：320 | 6  41  10  37  14 | 0.158（0.070，0.259）  0.073（0.033，0.166）  0.094（0.067，0.240）  0.080（0.052，0.202）  0.066（0.027，0.027） | -1.213  -1.360 | 0.234  0.173 |
