# Supplementary material for: The circular RNA hsa_circ_0045800 serves as a favorable biomarker in pathogenesis of sjögren's syndrome
Source: Clin Rheumatol. 2024 Jun 13;43(8):2585–94. doi: 10.1007/s10067-024-06999-0 (PMC11269352; doi:10.1007/s10067-024-06999-0)
Supplement: Supplementary file 4 — Supplementary file4 (DOCX 13 KB) [file 10067_2024_6999_MOESM4_ESM.docx]

**Supplyment Table 4**

| Index | n | hsa_circ_0045800  *M*（P25,P75） | Z value | *P* value |
| --- | --- | --- | --- | --- |
| Hypertension  Yes  No  Coronary heart disease  Yes  No  Hyperlipemia  Yes  No  Hyperuricemia  Yes  No  Fatty liver  Yes  No  Interstitial lung disease  Yes  No  Hypothyroidism  Yes  No  Chronic gastritis  Yes  No | 9  41  8  42  25  25  11  39  6  45  23  27  6  45  17  33 | 0.171（0.069，0.259）  0.022（0.022，0.049）  0.093（0.070，0.277）  0.027（0.011，0.048）  0.076（0.042，0.146）  0.076（0.040，0.218）  0.122（0.034，0.149）  0.071（0.066，0.190）  0.097（0.061，0.208）  0.071（0.040，0.181）  0.101（0.067，0.190）  0.060（0.034，0.143）  0.015（0.009，0.075）  0.078（0.049，0.202）  0.071（0.045，0.093）  0.087（0.031，0.233） | 0.079  -1.42  -0.243  -1.253  -0.663  -1.470  -2.251  -1.0342 | 0.081  0.160  0.808  0.210  0.529  0.142  0.022*  0.301 |
